# Evidence of the Immunomodulatory Effects of Selective Serotonin Reuptake Inhibitors in Patients With Depression Through a Systematic Review

**DOI:** 10.7759/cureus.62991

**Published:** 2024-06-23

**Authors:** Ankit N Patel, Jagdish Varma, Barna Ganguly

**Affiliations:** 1 Pharmacology, Pramukhswami Medical College, Karamsad, IND; 2 Psychiatry, Pramukhswami Medical College, Karamsad, IND

**Keywords:** immunomodulatory effect, depression, crp, tnf-α, il-6, antidepressant, ssri

## Abstract

Depression is a common illness, affecting >264 million people worldwide. According to the literature, depression patients have baseline subclinical inflammation. The immunomodulatory effects of antidepressants, such as selective serotonin reuptake inhibitors (SSRIs), are largely unclear and poorly understood. Using evidence-based medicine, this study aimed to determine the immunomodulatory effects of SSRIs by assessing changes in immunomodulatory markers following SSRI treatment. Using the PubMed website, a literature search was conducted with various terminologies related to the treatment of depression and various markers of inflammation. Out of 387 retrieved articles, after critical appraisal and screening based on inclusion and exclusion criteria, 17 were selected. Qualitative synthesis and quantitative analysis were carried out. RevMan 5 software was used to synthesize and evaluate the data. Microsoft Word and Excel (Microsoft Corporation, Redmond, Washington, United States) were used for generating tables and figures. We extracted data from a total of 839 patients in 17 studies. A highly significant reduction in interleukins-6 (IL-6) (standardised mean difference (SMD) = 1.32 (95% confidence interval (CI): 0.58, 2.06), Z = 3.48, P = 0.0005), a significant reduction in tumor necrosis factor-alpha (TNF-α) (SMD = 1.29 (95% CI: 0.19, 2.39), Z = 2.30, P = 0.02) but no change in overall C-reactive protein (CRP) (SMD = 0.40 (95% CI: -0.26, 1.07), Z = 1.19, P = 0.23) levels were observed by using the random-effects model. There was substantial heterogeneity found between the studies. SSRIs have an immunomodulatory effect in patients with depression by significantly reducing the peripheral pro-inflammatory cytokine markers of IL-6 and TNF-α, which may contribute to ameliorating the response to antidepressant drug treatment. In contrast, no effects of SSRIs on acute-phase protein CRP were found.

## Introduction and background

Depression, a common term used for a disorder called major depressive disorder (MDD), is a neurotic type of mood disorder [1]. Depression is a very common illness, with several affected people crossing 264 million worldwide. In 2015-16, the mental health of Indians was evaluated under the National Mental Health Survey, which revealed that around fifteen percent of adults in India have one or more mental health issues that may need active medical intervention, and per twenty Indians, at least one has suffered from depression [2].

In recent years, an increasing amount of evidence has been gathered suggesting that MDD may affect the immune system, which appears as a derangement in anti-inflammatory and pro-inflammatory cytokines [3,4]. The main cytokine markers of pro-inflammatory effect are interleukins 1β, 6 (IL-1β and IL-6, respectively), and tumor necrosis factor-alpha (TNF-α), while the main cytokine markers of anti-inflammatory effect include Interleukins 4, 10, 11, and 13 [5,6]. Evidence generated by the multiple studies shows that these cytokines of pro-inflammation like IL-6, TNF-α, and acute-phase reactant proteins like C-reactive protein (CRP) are increased in individuals with depression, and not only inflammatory changes are seen in depressive patients, but inflammation may contribute as one of the etiological factors in depression [7-9].

There are many treatments, like psychosocial therapy, pharmacotherapy, electroconvulsive therapy, and other novel therapies like phototherapy, vagal nerve stimulation, sleep deprivation therapy, etc., available to treat depression [1]. Current pharmacotherapy for depression targets mostly monoamines like serotonin (5-HT) and norepinephrine (NA), intending to improve the level of monoamines in neuronal synapses to ameliorate depression. Evidence shows that drugs from the tricyclic antidepressants (TCAs) and serotonin noradrenaline reuptake inhibitors (SNRIs) groups modulate both serotonergic and noradrenergic neurons, while drugs from the selective serotonin reuptake inhibitors (SSRIs) group modulate only serotonergic neurons selectively [1]. Due to fewer side effects and effectiveness similar to TCAs, SSRIs like fluoxetine, citalopram, escitalopram, paroxetine, sertraline, etc. are currently used as first-line therapy in depression [1].

Although some clinical studies and meta-analyses showed that SSRIs and TCAs reduce blood levels of several markers of inflammation in patients with depression, other clinical and meta-analysis studies have concluded that the use of antidepressants may lead to an increase in inflammatory markers or does not have any significant effect [10,11].

In short, individuals suffering from depression exhibit an indivisible relationship between their brain and immune system. However, available data are unclear and inconclusive in the direction of what exact interaction is happening and with what drug-inflammatory pathway or inflammatory marker is affected, as there are discrepancies in the results of available scientific studies.

As there is a significant lack of clarity and a gap in the understanding of the immunomodulatory effect of antidepressant drugs, including SSRIs, this study aimed to find out the immunomodulatory effect of four major SSRIs, escitalopram, sertraline, fluoxetine, and paroxetine, used in clinical practice, by evaluating changes in immunomodulatory markers (IL-6, TNF-α, and CRP).

## Review

Methods

The published research articles were systematically reviewed and analyzed in the current systematic review. The study was carried out after approval from the institutional ethics committee. The study method was adapted from Cochrane's guidelines for systematic reviews; also, the book "Finding What Works in Health Care: Standards for Systematic Reviews" was also referred to for information related to study methodology [12,13]. The present study was carried out in compliance with the 2020 revised standards of Preferred Reporting Items for Systematic Reviews and Meta-Analyses (PRISMA) [14].

Source of Data

Research articles were searched on the internet in the database of MEDLINE, that is PubMed.

Inclusion Criteria

Data of patients of either sex, aged ≥18 years, who were either newly diagnosed cases of major depressive disorder or who were off anti-depression treatment for at least three months (or five half-lives) from full-text English articles, either interventional or observational, with evidence on the effect of four major SSRIs, escitalopram, sertraline, fluoxetine, and paroxetine, on serum levels of IL-6 and/or TNF-α and/or CRP from the PubMed database were included.

Exclusion Criteria

Excluded articles included information on the effects of antidepressant medications other than escitalopram, sertraline, fluoxetine, and paroxetine; non-English articles; articles with closed or paid access to data, except articles accessible through the institute's central library; and articles with only an abstract and no full text.

Sampling Procedures

**Figure 1:**
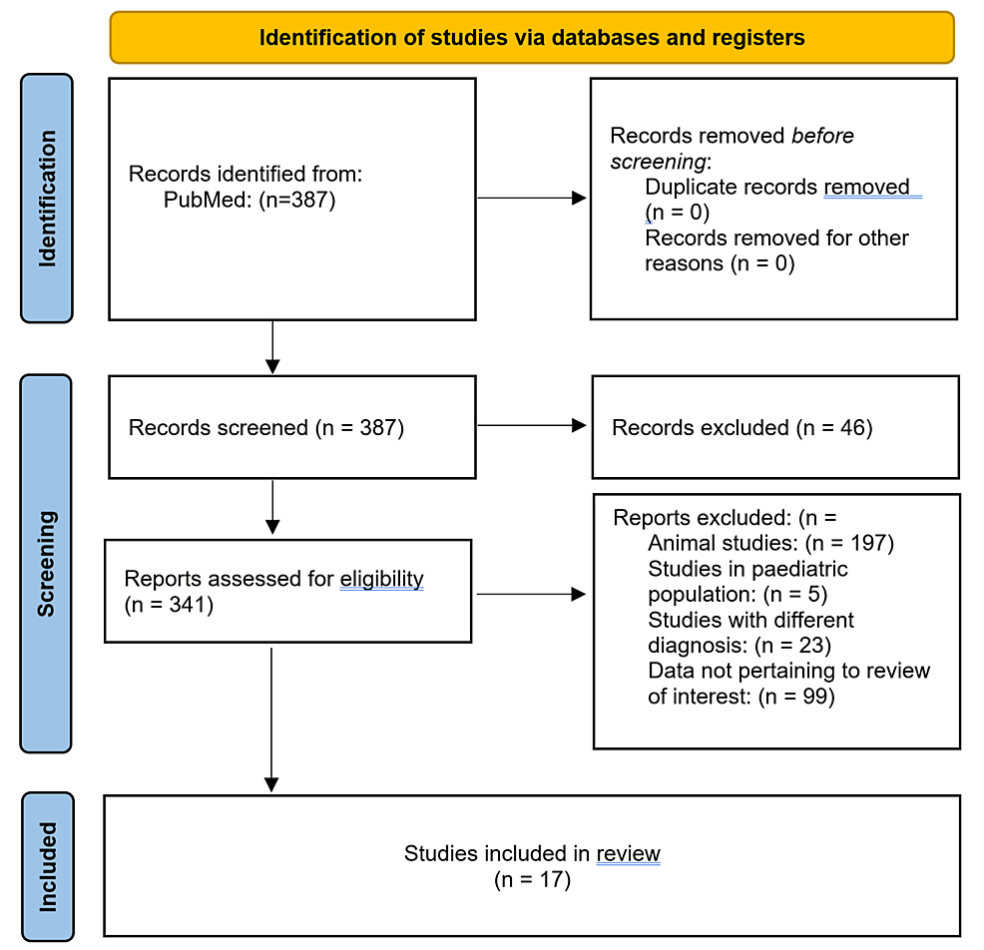
PRISMA flowchart depicting the selection of studies according to eligibility criteria PRISMA: Preferred Reporting Items for Systematic Review and Meta-Analysis

Research studies were searched on PubMed using the following search strategy: (Escitalopram OR Fluoxetine OR Sertraline OR Paroxetine OR Selective Serotonin Reuptake Inhibitor OR SSRI) AND (C-Reactive Protein OR C Reactive Protein OR CRP OR IL-6 OR Interleukin 6 OR Interleukin-6 OR IL6 OR TNF-alpha OR TNF alpha OR Tumor necrosis factor α OR tumor necrosis factor alpha OR TNF-α OR TNF α) AND (Major Depressive Disorder OR Depression OR Major Depression). Following a thorough and critical scientific evaluation of the research, those that met the inclusion and exclusion criteria were chosen to be included in the final analysis (Figure 1). After that, information was taken from the included research and entered into an Excel sheet (Microsoft Corporation, Redmond, Washington, United States).

The risk of bias was also evaluated for each included study. The quality and risk of bias of the included randomized clinical studies were assessed using the criteria established by the Cochrane collaboration [15]. Additionally, the Newcastle-Ottawa scale for observational studies, which was the basis for the method described by Liu et al., 2019, was used to evaluate all included research articles [16,17].

Study Design

Initially, we assessed the qualitative (qual) part, followed by the rest of the quantitative (QUAN) analytical part. Qual (descriptive qualitative part): The free listing exercise for the selected studies was done. Descriptive analysis was done by generating items manually. The items identified were the type and design of the study, the risk of bias assessment, confounding factors in a study, changes in biomarkers and the depression rating scale (DRS), and the limitations of the included study. After reviewing the studies qualitatively, we proceeded with a quantitative (QUAN) analysis of the studies.

Statistical Analysis

Review Manager (RevMan) 5 software was used for data synthesis and evaluation to determine the immunomodulatory effects of SSRIs [18]. A portion of the data was in the median, whereas the majority of the extracted values were in the form of the mean. The values from the median ± IQR were transformed to mean ± SD. The generic inverse variance approach in RevMan 5 was utilized to pool the standardized mean difference (SMD), which was selected as the summary statistic for the meta-analysis. Due to the heterogeneity of the included studies and their wide range of data variances, a random effects model was selected. P values less than 0.05 and less than 0.001 were regarded as highly significant and statistically significant, respectively. Publication bias was evaluated in RevMan 5 by charting the effect size against the sample size for each study's data using a funnel plot. Heterogeneity in variations in inflammatory cytokine levels was visually evaluated from the forest plot of the SMD of individual studies. The I^2^ heterogeneity statistic was used in RevMan-5 software to evaluate statistical estimates of heterogeneity.

Results

Demographic Details of Included Studies

Seventeen studies [8,19-34] fulfilled the inclusion criteria. From these 17 studies, data on a total of 843 participants were extracted. Demographic details, including sample size, gender distribution, age range, and country of origin of each study, as well as details of the antidepressant SSRI used and the biomarkers assessed, are described in Table 1.

**Table 1:** Demographic details of included studies TNF-α: tumor necrosis factor-alpha; CRP: C-reactive protein; SSRI: selective serotonin reuptake inhibitors; RCT: randomized controlled trial

Study name	Type of study (RCT/Non-RCT)	Sample size	Male	Female	Age range	Country of origin	Biomarker assessed	Antidepressant used	Dose (mg/day)	Duration of treatment
Mao 2022 [8]	Non-RCT	40	13	27	18-60	Han	CRP, IL-6	SSRI	-	6
Brunoni 2018 [19]	RCT	87	30	57	18-75	Brazil	IL-6	Escitalopram	10	10
Brunoni 2014 [20]	RCT	18	7	11	18-75	Brazil	IL-6, TNF-α	Sertraline	50	6
Rawdin 2013 [21]	Non-RCT	20	7	13	>18	USA	IL-6	Sertraline	50-200	8
Lavretsky 2011 [22]	RCT	37	15	22	> 60	USA	CRP	Escitalopram	10-20	10
Zhou 2022 [23]	Non-RCT	71	21	50	18-65	China	CRP	Escitalopram	10	12
Sutcigil 2007 [24]	Non-RCT	23	12	11	>18	Turkey	TNF-α	Sertraline	50-100	8
Abdallah 2020 [25]	RCT	40	22	18	23-57	Egypt	CRP, IL-6, TNF-α	Fluoxetine	20	12
Abdallah 2021 [26]	RCT	40	9	31	20-60	Egypt	TNF-α	Escitalopram	20	6
Simon 2021 [27]	RCT	23	-	-	18-60	USA	TNF-α	Sertraline	-	6
Chavda 2011 [28]	RCT	96	-	-	15-55	India	CRP	Escitalopram, Fluoxetine	20	8
Abbasi 2012 [29]	RCT	20	14	6	18-50	Iran	IL-6	Sertraline	200	6
Jazayeri 2009 [30]	RCT	14	4	10	20-59	Iran	IL-6	Fluoxetine	20	8
Dong 2021 [31]	Non-RCT	104	33	71	18-72	China	IL-6	Paroxetine	10-40	8
Eller 2008 [32]	Non-RCT	100	35	65	>18	Estonia	TNF-α	Escitalopram	10-20	12
Chen 2018 [33]	Non-RCT	50	-	-	20-65	Taiwan	IL-6, TNF-α	Paroxetine	10-40	8
Liu 2015 [34]	RCT	60	30	30	18-60	China	IL-6	SSRI	-	6

Quality and Risk of Bias Assessment of Included Studies

We conducted an assessment of the quality and risk of bias of the included randomized clinical trial. Selection bias, performance bias, detection bias, and attrition bias were the categories used to assess the risk of bias and quality of ten randomized clinical trials (RCTs) based on Cochrane’s guidelines.

Selection bias: All the studies used one or another type of method to generate a random sequence, or randomization. The risk of bias in allocation concealment was high in two studies, with unclear information in one study while all others clearly described the method of allocation concealment.

Performance bias: One study described blinding unclearly; one study has not blinded participants or investigators; and one study blinded only investigators and not participants due to the type of intervention they were comparing, while the rest of all studies blinded both participants and investigators.

Detection bias: When blinding of outcome assessment is not done, this bias is likely to happen. Three studies have an unclear description of blinding of outcome assessment; four studies have not blinded outcome assessment; and three have implemented it.

Attrition bias: Occurs when there is incomplete outcome data that is not taken care of or considered. Only two out of 10 studies have not described incomplete outcome data, while the rest of the studies have taken care of incomplete outcome data.

The overall risk of bias and quality assessment: According to this evaluation, out of ten studies, five had a low or minimum bias risk and were regarded as good-quality research, four had a medium risk and were regarded as average-quality research, and one had a high bias risk and was regarded as low-quality research.

**Figure 2:**
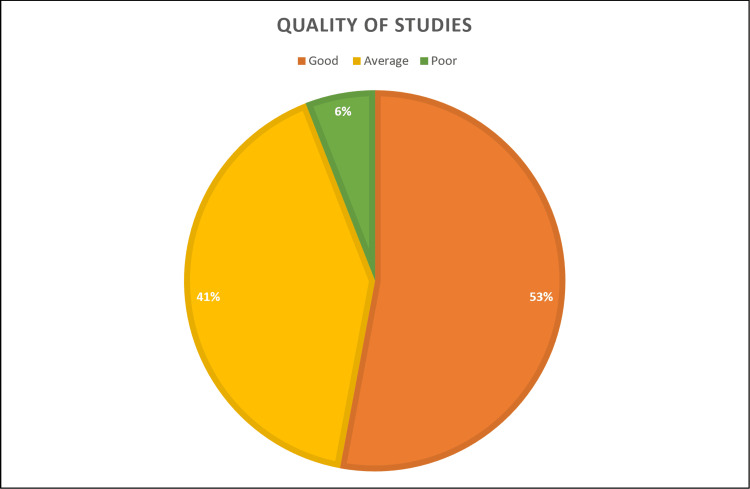
Assessing the quality of studies

Assessing quality and risk of bias based on the method described by Liu et al., 2019 [17]: Based on this assessment, out of 17, nine studies had a low bias risk and were considered good-quality research. Seven studies had a medium bias risk and were considered average-quality research, while a single study had a high bias risk and was considered poor-quality research (Figure 2).

Qualitative (Qual) Synthesis

Items identified for synthesis were study type and design: the majority were randomized controlled blinded studies, and one-third of the total studies were nonrandomized with both blinding and partially blinding methods; risk of bias assessment: studies with RCTs showed a lesser risk of bias while others showed an acceptable risk of bias; confounding factors: they were mainly related to age, gender, body mass index (BMI), tobacco consumption, and some certain co-morbid diseases that didn’t call for a major change in the outcome; changes in the biomarkers and depression rating scale: the majority of the studies showed a reduction in the biomarkers and an improvement in the score of the depression rating scale; limitations of the studies: some of the studies mentioned smaller sample sizes, concomitant co-morbidities, confounders of smoking, and BMI. These findings were found to be relevant and appropriate for further quantitative analysis of this evidence-based study.

Quantitative Analysis of Data (QUAN)

**Figure 3:**
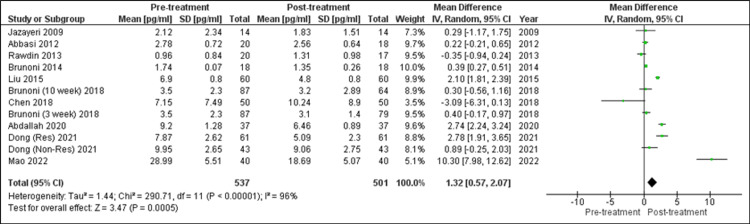
Forest plot of overall change in IL-6 level after SSRI treatment [8,19-21,25,29-31,33,34] 10 week: 10 weeks of treatment duration; 3 week: 3 weeks of treatment duration; res: responding to SSRI; non-res: not responding to SSRI; SSRI: selective serotonin reuptake inhibitor With the use of the random-effects model, after treatment with SSRI, statistically highly significant reduction in IL-6 level (p = 0.0005) was observed.

Changes in IL-6: A total of 10 research studies analyzed the effect of SSRI on IL-6 levels, and in the current analysis, they were analyzed to determine the changes produced in IL-6 levels after such treatment (Figure 3). Brunoni et al. (2018) measured IL-6 levels at three and 10 weeks post-treatment. So, both values had been considered separately [19]. Dong et al. (2011) described data in responders (Res) and non-responders (Non-Res) groups [31]. So, the values of both groups were considered separately. An observation of a statistically highly significant decrease in IL-6 level was made using the random-effects model. (SMD = 1.32 (95% confidence interval (CI): 0.57, 2.07), Z = 3.47, P = 0.0005). The research studies were to be quite heterogeneous. (τ^2^ = 1.44, χ^2^ = 290.71, df = 11, p < 0.00001, I^2^ = 96%).

**Figure 4:**
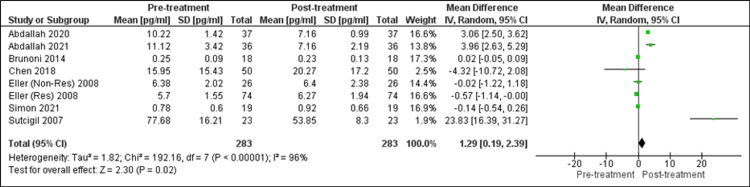
Forest plot of overall change in TNF-α level after SSRI treatment [20,24-27,32,33] res: responding to SSRI; non-res: not responding to SSRI; SSRI: selective serotonin reuptake inhibitor; TNF-α: tumor necrosis factor-alpha With the use of the random-effects model, after treatment with SSRI, statistically significant reduction in TNF-α level (p = 0.02) was observed.

Changes in TNF-α: Seven research studies in total measured the effect of SSRI on TNF-α levels, and in the current analysis, they were analyzed to determine the changes produced in TNF-α levels after such treatment (Figure 4). Eller et al. (2008) described data in responders (Res) and non-responders (Non-Res) groups. So, the values of both groups had been considered separately [32]. An observation of a statistically significant decrease in TNF-α level was made using the random-effects model. (SMD = 1.29 (95% CI: 0.19, 2.39), Z = 2.30, P = 0.02). These research studies were also to be quite heterogeneous. (τ^2^ = 1.82, χ^2^ = 192.16, df = 7, p < 0.00001, I^2^ = 96%).

**Figure 5:**
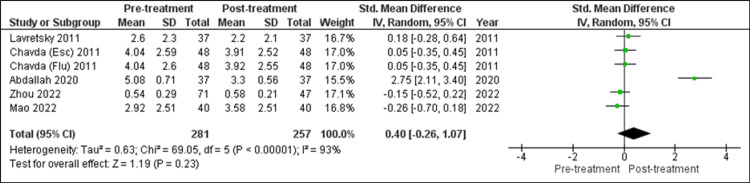
Forest plot of overall change in CRP level after SSRI treatment [8,22,23,25,28] esc: escitalopram groupl; flu: fluoxetine group; SSRI: selective serotonin reuptake inhibitors; CRP: C-reactive protein With the use of the random-effects model, after treatment with SSRI, statistically no change in CRP level (p = 0.23) was observed.

Note: In forest plots (Figures 3, 4, 5), each “square” shows the effect size for a single study, with the horizontal line running through each square demonstrating the width of the 95% CI. The size of the square is proportional to the weight attributed to each study. The “diamond” represents the summary effect size, with the middle equaling the summary effect size and the width depicting the width of the overall 95% CI.

Changes in CRP: A total of five research studies analyzed the effect of SSRI treatment on CRP levels and were analyzed in the current research for the change in CRP level after such treatment (Figure 5). Chavda et al. (2011) carried out a study for the escitalopram (Esc) and fluoxetine (Flu) groups. So, the values of both groups had been considered separately [28]. An observation of no statistically significant change in CRP level was made using the random-effects model. (SMD = 0.40 (95% CI: -0.26, 1.07), Z = 1.19, P = 0.23). Like other studies, these five research studies were also to be quite heterogeneous. (τ^2^ = 0.63, χ^2^ = 69.05, df = 5, p < 0.00001, I^2^ = 93%).

**Figure 6:**
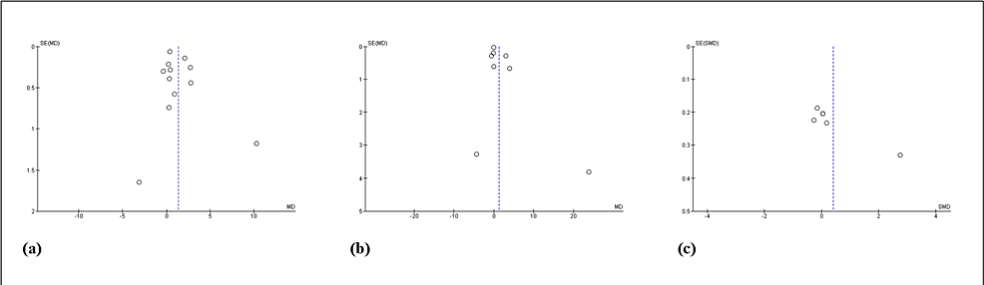
Funnel plots of overall changes in IL-6 (a), TNF-α (b), and CRP (c) levels after SSRI treatment SE(MD) or SE(SMD): standard error of mean difference (precision); MD or SMD: mean difference (effect size); SSRI: selective serotonin reuptake inhibitors; TNF-α: tumor necrosis factor-alpha; CRP: C-reactive protein Funnel plots of overall changes in IL-6, TNF-α and CRP levels after SSRI treatment were visually asymmetric.

Funnel plots: Because of limited study impact and heterogeneity in the study population, dose, and length of therapy, funnel plots of overall changes in peripheral cytokines IL-6, TNF-α, and CRP levels following SSRI treatment were visually asymmetric (Figure 6).

Discussion

IL-6

In our analysis, out of 17 studies, 10 included IL-6 for their analysis, which cumulatively showed a significant lowering of IL-6 levels after treatment with SSRIs. The results of our findings on IL-6 are similar to those of one of the systematic reviews of 15 studies by Wang et al., 2019 [35]. Likewise, a meta-analysis conducted by Więdłocha et al. (2018) and by Hiles et al. (2012) to examine how antidepressants affect peripheral cytokines also showed a decrease in cytokine IL-6 levels [36,37]. In 2011, Hannestad et al. carried out a meta-analysis of 22 studies and found that antidepressant medications lower blood IL-6; stratified subgroup analysis indicated that SSRIs, but not other antidepressants, lowered IL-6 levels [38]. On the contrary, a systematic review carried out by Liu et al. in 2020 showed no significant effect on IL-6 after antidepressant treatment [17]. Similarly, a systematic review carried out by Almeida et al. in 2020 showed unchanged IL-6 levels in fluoxetine-treated depressive patients [39]. A study by Basterzi et al. (2005), carried out on 23 patients, showed that SSRI treatment reduces IL-6 levels significantly [40]. On the contrary, a study by Kubera et al. (2004) showed the opposite result [41].

TNF-α

Out of 17 studies in our analysis, seven included TNF-α for their analysis, which cumulatively showed a significant reduction of TNF-α level after treatment with SSRIs. The results of our findings on TNF-α are like a systematic review, which included 22 studies by Wang et al. (2019) [35]. Similarly, a systematic review by Liu et al. (2020) also showed a reduction in the level of TNF-α after treatment with antidepressants [17]. Recent systematic reviews, one by Almeid et al., 2020, and another by García-García et al., 2022, intended to find out the immunomodulatory effect of fluoxetine, showed that TNF-α levels were reduced after treatment [39,42]. On the contrary, separate systematic reviews, one by Więdłocha et al. (2018), which included 32 studies, and another by Hannestad et al. (2011), evaluated the immunomodulatory effect of antidepressant drugs in patients with depression and showed no significant effect on TNF-α level [37,38]. Although a study by Kubera et al. in 2004 showed no effect of antidepressant drugs on TNF-α level [41], three different studies by Yoshimura et al. (2009), Gupta et al. (2017), and Tuglu et al. (2003) showed a decreased level of TNF-α [43-45].

CRP

We found that out of 17 studies, five included CRP for their analysis, which cumulatively showed no significant change in CRP level after treatment with SSRIs, which is comparable to the outcomes of Więdłocha et al., 2018 [37] and a systematic review by Liu et al., 2020 [17]. Conversely, a systematic review of a total of eight studies by Hiles et al. (2012) showed a significant reduction in CRP levels after treatment with antidepressants [36].

Limitations

A few PubMed-indexed full-text articles could not be captured for various reasons. A few non-randomized controlled trials and observational studies were also included. These studies have a higher risk of multiple biases, which can affect the overall result of this review. Due to the variability in the data, a high level of heterogeneity in the research methodology and study population was found. Some confounding factors, like comorbidities, inflammatory conditions, age, and gender, may affect the overall study results.

## Conclusions

In depressive patients, selective serotonin reuptake inhibitors have an immunomodulatory effect by notably lowering pro-inflammatory peripheral cytokines IL-6 and TNF-α, which may also contribute to ameliorating the response of antidepressant drug treatment. In contrast, we found no effect on the acute-phase protein CRP. These results imply that the relationship between the antidepressant drug class of selective serotonin reuptake inhibitors and the immune system is nuanced and calls for more investigation.
